# Fetal Programming by Methyl Donors Modulates Central Inflammation and Prevents Food Addiction-Like Behavior in Rats

**DOI:** 10.3389/fnins.2020.00452

**Published:** 2020-06-03

**Authors:** Gabriela Cruz-Carrillo, Larisa Montalvo-Martínez, Marcela Cárdenas-Tueme, Sofia Bernal-Vega, Roger Maldonado-Ruiz, Diana Reséndez-Pérez, Dalia Rodríguez-Ríos, Gertrud Lund, Lourdes Garza-Ocañas, Alberto Camacho-Morales

**Affiliations:** ^1^Department of Biochemistry, College of Medicine, Universidad Autónoma de Nuevo León, San Nicolás de los Garza, Mexico; ^2^Neurometabolism Unit, Center for Research and Development in Health Sciences, Universidad Autónoma de Nuevo León, San Nicolás de los Garza, Mexico; ^3^Department of Cell Biology and Genetics, College of Biological Sciences, Universidad Autónoma de Nuevo León, San Nicolás de los Garza, Mexico; ^4^Department of Genetic Engineering, CINVESTAV Irapuato Unit, Irapuato, Mexico; ^5^Department of Pharmacology and Toxicology, College of Medicine, Universidad Autónoma de Nuevo León, San Nicolás de los Garza, Mexico

**Keywords:** programming, methylome, addiction, nucleus accumbens, inflammation

## Abstract

Fetal programming by hypercaloric intake leads to food addiction-like behavior and brain pro-inflammatory gene expression in offspring. The role of methylome modulation during programming on central immune activation and addiction-like behavior has not been characterized. We employed a nutritional programming model exposing female Wistar rats to chow diet, cafeteria (CAF), or CAF-methyl donor’s diet from pre-pregnancy to weaning. Addiction-like behavior in offspring was characterized by the operant training response using Skinner boxes. Food intake in offspring was determined after fasting–refeeding schedule and subcutaneous injection of ghrelin. Genome-wide DNA methylation in the nucleus accumbens (NAc) shell was performed by fluorescence polarization, and brain immune activation was evaluated using real-time PCR for pro-inflammatory cytokines (IL-1β, TNF-1α, and IL-6). Molecular effects of methyl modulators [S-adenosylmethionine (SAM) or 5-azatidine (5-AZA)] on pro-inflammatory cytokine expression and phagocytosis were identified in the cultures of immortalized SIM-A9 microglia cells following palmitic acid (100 μM) or LPS (100 nM) stimulation for 6 or 24 h. Our results show that fetal programming by CAF exposure increases the number of offspring subjects and reinforcers under the operant training response schedule, which correlates with an increase in the NAc shell global methylation. Notably, methyl donor’s diet selectively decreases lever-pressing responses for reinforcers and unexpectedly decreases the NAc shell global methylation. Also, programmed offspring by CAF diet shows a selective IL-6 gene expression in the NAc shell, which is reverted to control values by methyl diet exposure. *In vitro* analysis identified that LPS and palmitic acid activate IL-1β, TNF-1α, and IL-6 gene expression, which is repressed by the methyl donor SAM. Finally, methylation actively represses phagocytosis activity of SIM-A9 microglia cells induced by LPS and palmitic acid stimulation. Our *in vivo* and *in vitro* data suggest that fetal programming by methyl donors actively decreases addiction-like behavior to palatable food in the offspring, which correlates with a decrease in NAc shell methylome, expression of pro-inflammatory cytokine genes, and activity of phagocytic microglia. These results support the role of fetal programming in brain methylome on immune activation and food addiction-like behavior in the offspring.

## Introduction

Maternal obesity or maternal hypercaloric intake in humans and murine models is associated with an increased risk of systemic and central pathologies early in life. We and others have published that maternal overnutrition programs negative metabolic and immune profiles (Cardenas-Perez et al., 2018; Maldonado-Ruiz et al., 2019), and addiction (Camacho, 2017; Sarker et al., 2018), as well as depression-like behavior phenotypes in the offspring (de la Garza et al., 2019). Excessive high-energy food consumption seems to modulate positively or negatively a hedonic phenotype in humans and animal models. Several neurotransmitter-related hypotheses were proposed to explain unhealthy eating; for instance, the hypothesis of the reward deficiency states that in the context of high caloric overfeeding or obesity, uncontrolled food intake is activated in order to compensate for a deficient reward effect of food consumption due to failure in the dopaminergic activity (Kenny, 2011; Land and DiLeone, 2012; Barry et al., 2018; Courtney et al., 2018; Gold et al., 2018; van Galen et al., 2018; DiFeliceantonio and Small, 2019; Ducrocq et al., 2019). Under this scenario, impulsiveness for unhealthy eating and caloric overconsumption as a reward is developed (Volkow et al., 2011; Dietrich et al., 2014; Bongers et al., 2015). Classically, reward and incentive salience are integrated to the nucleus accumbens (NAc), whereas preoccupation/anticipation including craving, impulsivity, and executive function involve the prefrontal cortex (PFC) (Volkow and Wise, 2005; Volkow et al., 2013; Koob and Volkow, 2016).

Potential molecular or cellular mechanism leading to addiction-like behavior during maternal hypercaloric exposure is not totally understood. Hypercaloric diets themselves stimulate central and peripheral inflammatory nodes. For instance, exposure to saturated fatty acids during pregnancy activate the Toll-like type 4 receptor signaling (TLR4) in adipocytes and macrophages (Shi et al., 2006) and promotes substantial activation of microglia in the hypothalamus (Maldonado-Ruiz et al., 2019). Systemic and central immune activation pathways regulate abnormal behaviors including depression (Wohleb et al., 2016), schizophrenia (Yuan et al., 2019), and addiction (Alfonso-Loeches et al., 2010; Pascual et al., 2011; Hofford et al., 2018). Conversely, systemic treatment of the anti-inflammatory drug minocycline to methamphetamine-addicted rats reverted addiction-like behavior (Snider et al., 2013; Attarzadeh-Yazdi et al., 2014). Of note, morphine binds to the TLR4 directly, which increases the risk of drug-induced reinstatement (Schwarz and Bilbo, 2013), and blocking the TLR4 pathway in the VTA of rats reduces cocaine and morphine-primed drug seeking (Tanda et al., 2016; Chen et al., 2017; Brown et al., 2018), which seems to depend on microglia activation (Northcutt et al., 2015). Physiologically, TLR4 signaling regulates glutamatergic stimulation in the NAc shell (Kashima and Grueter, 2017); however, repeated cocaine administration activates striatal microglia, leading to TNF-α production and disrupting glutamatergic synaptic strength in the NAc shell (Lewitus et al., 2016). These data suggest that microglia activation and TLR4 signaling positively modulate reinstatement for drug-seeking behavior. It is unknown if addiction-like behavior primed by maternal hypercaloric programming sets a TLR4-like inflammatory profile in NAc capable of regulating addiction-like behavior in the offspring.

Exposure to hypercaloric diet in mice sets a higher proliferation and immune response of myeloid progenitor cells by activating an epigenetic reprogramming mechanism (Christ et al., 2018), known as trained immunity (Netea, 2013). Physiologically, epigenetic modulation, such as DNA methylation, positively activates neuronal differentiation in mammals (Mohn et al., 2008), regulates synaptic plasticity in the hippocampus (Levenson et al., 2006), and, by its own neuronal activity induced by external cues, closely modulates DNA methylation (Maag et al., 2017). For instance, chronic exposure to amphetamine, ecstasy, or MDMA favors a positive global DNA methylation and pro-inflammatory profile in the NAc shell of rats (Mychasiuk et al., 2013), and shows an increase in tri-methylation of lysine 4 in histone H3 (H3K4me3) on the pronociceptin, prodynorphin, and neuropsin promoters, while decreasing the acetylation of lysine 9 in Histone H3 (H3K9ac) in the nociceptin/orphaninFQ (pN/OFQ) (Caputi et al., 2016). Also, maternal care of rat offspring blocks drug-induced reinstatement of morphine by decreasing the methylation of the IL-10 promoter in NAc shell (Schwarz et al., 2011). These evidences support a potential role of the brain proinflammatory profile and drug addiction susceptibility actively modulated by methylation and/or acetylation of histones and/or DNA. Overall, we hypothesized that maternal programming by caloric diet exposure primes DNA methylation changes and a pro-inflammatory profile in NAc favoring addiction-like behavior for natural rewards in the offspring.

The present study aimed to explore the role of maternal nutritional programming and its effects on DNA methylation, pro-inflammatory profile, and susceptibility to addiction-like behavior in the offspring.

## Materials and Methods

### Animals and Housing

All the experiments were performed using 2-month-old wild-type female Wistar rats (initial body weight, 200–250 g). Animals were handled according to the NIH Guide for the Care and Use of Laboratory Animals (NIH Publications No. 80–23, revised in 1996). We followed the Basel Declaration to implement the ethical principles of Replacement, Reduction, and Refinement of experimental animal models. Our study was approved by the local Animal Care Committee (BI0002) at the Universidad Autónoma de Nuevo León, Mexico. Rats were housed individually in Plexiglas-style cages, maintained at 20–23°C in a temperature-controlled room with a 12-h light/dark cycle. Water was available *ad libitum* in the home cage. Food availability is described below.

### Diets

• The standard chow diet formula contained 57% carbohydrates, 13% lipids, and 30% proteins, and 290 mg of sodium, caloric density = 3.35 kcal/g (LabDiet, St. Louis, MO 63144, 5001, United States). Cafeteria (CAF) diet was made of liquid chocolate, biscuits, bacon, fried potatoes, standard chow diet, and pork paté at a 1:1:1:1:1:1:2 ratio, including 39% carbohydrates, 49% lipids, 12% proteins, and 290 mg of sodium, caloric density = 3.72 kcal/g, as we reported (Camacho, 2017; Cardenas-Perez et al., 2018; Maldonado-Ruiz et al., 2019). CAF diet with methyl donors consisted of CAF formula enriched with betaine (5 g/kg of diet), choline (5,37 g/kg of diet), folic acid (5,5 mg/kg of diet), and vitamin B12 (0.5 mg/kg of diet). Total fiber available for CAF diet and standard chow diet is similar and can be found in Supplementary Table S1 in Supplementary Material.

### Maternal Programming Model

**FIGURE 1:**
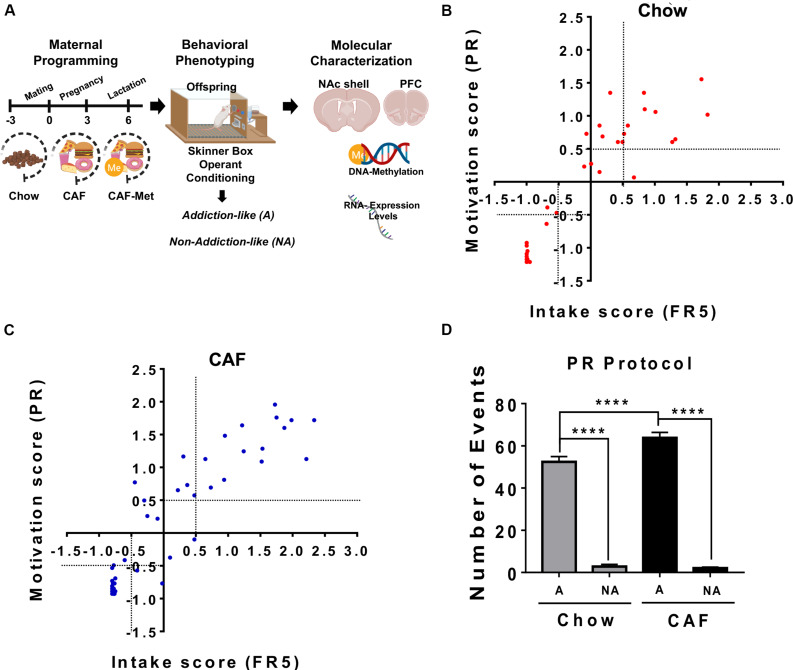
Maternal programming by CAF diet primes addiction-like behavior in offspring. **(A)** Maternal programming model. We fed female Wistar rats for 9 weeks including pre-pregnancy, pregnancy, and lactation with standard chow diet (C, *n* = 8) or CAF diet (*n* = 14). Offspring was fed with control diet after weaning until 2 months of age and was trained in the operant training test protocol to diagnosed non-addiction and addiction-like behavior. Molecular characterization of pro-inflammatory expression and global DNA methylome in the NAc shell were performed in non-addiction and addiction-like behavior subjects. **(B,C)** To diagnose addiction-like and non-addiction-like behavior phenotypes in the offspring subjects, we calculated the intake and motivation scores by integrating lever presses during the FR5 and PR schedules, respectively, as reported (Bock et al., 2013; Le et al., 2017). We identified addiction-like and non-addiction-like behavior in the offspring by the FR5/PR ratio by setting a +0.5 or –0.5 score at “*x*” and “*y*” axis to characterize the addiction or non-addiction-like behavior subjects, respectively. Motivation (based on PR) or intake (based on FR5) scores in the offspring programmed by chow or CAF, respectively. Graphs show mean ± SEM. Chow, *n* = 53; CAF, *n* = 36. **(D)** Number of events during PR protocol of subjects exposed to chow or CAF diet during programming. Graphs show mean ± SEM. Chow diet programmed, addict = 10, non-addict = 14; CAF diet programmed, addict = 10, non-addict = 27. Statistical significance after using one-way ANOVA, followed by a Bonferroni’s *post hoc* test. The differences between the groups of operant training test were tested by two-way repeated measures ANOVA. ****p < 0.0001. A, Addiction-like; NA, Non-addiction-like.

Animals were acclimated to the animal facility 7 days before being exposed to the diet. A total of 27 10-week-old female Wistar rats (initial body weight, 200–250 g) were housed in standard conditions as described above with *ad libitum* access to food and water. Females were randomized into three different dietary groups: standard chow diet (C, *n* = 8), CAF diet (*n* = 14), and CAF + methyl donor supplemented (CAF + Met, *n* = 5) and were exposed *ad libitum* to them for 9 weeks, including 3 weeks before mating, 3 weeks during gestation, and 3 weeks during lactation. Rats were mated with age-matched Wistar males for 2 days, after which males were removed from the home cage. Pregnancy diagnosis was performed after mating by vaginal plug. Female rats lacking plugs were returned to the home cage for a second mating. Pregnant rats were transferred to individual cages and were kept on the same diet until birth and lactation. Pregnant females might have uneven litter load during gestation; however, litters were adjusted to 10 pups per mother after birth. After 21 days of lactation, male offspring were exposed to a control diet until 2 months of age before the operant training test protocol (see below for details and Figure 1A for experimental design). We chose male offspring based on the potential hormone sensitive-behavioral effects in females. In any case, in order to follow the ethical principles of Replacement, Reduction, and Refinement of experimental animal models, we allocated all female offspring to a second experimental behavioral protocol, which is currently under investigation.

### Operant Training Test

We followed the operant training protocol using the Skinner box as reported before (Camacho, 2017), with slight modifications.

#### Rat Acclimation and Food Restriction

Upon arrival, offspring rats from the three dietary groups: chow diet, CAF, and CAF + Met were group-housed for at least 7 days to acclimatize with *ad libitum* access to standard chow diet and water. After acclimation, rats were subsequently single-housed, and food was restricted by lowering to 70% their daily chow food intake until they lost 5–10% of total body weight at 7 days. This manipulation allowed acquisition of efficient lever-press responding during training. Upon reaching desired weight, we adjusted daily food intake to stabilize their weights for the remainder of the training period.

#### Fixed Ratio (FR)-1 Schedule

Rats were trained to press the lever on a fixed ratio (FR)-1 reinforcement schedule where a single lever press delivers a food pellet to the receptacle. Only one lever is designated as “active,” showing a 5-s timeout (TO) to the FR1 schedule (FR1/TO-5), during which additional lever pressing does not result in the delivery of a food pellet. Each FR training session lasts 1 h or when 100 pellets have been delivered. Training on the FR1 schedule lasted 3 days.

#### Fixed Ratio (FR)-5 Schedule

We established the FR5/TO-5 seconds schedule where five active lever presses triggered the delivery of the food pellet. Training on the FR5 schedule lasted 4 days. As for FR1/TO-5, schedule rats were exposed to *ad libitum* access to standard chow and water.

#### Progressive Ratio (PR) Schedule

The PR testing was calculated as per Richardson and Roberts (1996) using the following formula (rounded to the nearest integer): = [5e (R × 0.2)]−5, where R is equal to the number of food rewards already earned plus 1 (i.e., next reinforcer). Thus, the number of responses required to earn a food reward follow the order: 1, 2, 4, 6, 9, 12, 15, 20, 25, 32, 40, 50, 62, 77, 95, and so on. The PR session lasts a maximum of 1 h per day. Failure to press the lever in any 10-min period results in termination of the session. We also verified performance on the PR schedule by documenting the stable reinforcement for food when the number of rewards earned in a 1-h session deviates by ≤10% for at least 3 consecutive days. Like in the FR1/TO-5 and FR5/TO-5 schedule, rats were exposed to *ad libitum* access to standard chow and water. Training on the PR schedule lasted 5 days.

#### Calculation of Motivation and Intake Scores

Addiction-like and non-addiction-like behavior in the offspring subjects was diagnosed by integrating lever presses during the FR5 and PR schedules, as reported (Bock et al., 2013; Le et al., 2017). We use the $\times =\frac{X_{i}-\bar{X}}{s.d}$, where *X*_i_ is the behavior value for each rat, $\bar{X}$ is the mean behavior value for all the rats, and *s.d.* is the standard deviation of the population behavior value. We identified addiction-like and non-addiction-like behavior in the offspring by the FR5/PR ratio by setting a +0.5 or −0.5 score at “*x*” (intake score) and “*y*” (motivation score) to characterize the addiction- or non-addiction-like behavior subjects, respectively. This analysis allows one to set the intake and motivation scores for each subject to classify them as high and low responses (addiction- or non-addiction-like behavior) to operant training for natural rewards during the FR5 and PR schedules.

Dams after lactation and offspring after addiction-like and non-addiction-like behavior characterization were sacrificed using decapitation according to the NORMA Oficial Mexicana NOM-062-ZOO-1999. We used decapitation to preserve DNA/RNA integrity. Also, we allocated all female offspring to a second experimental behavioral protocol, which is currently under investigation.

### Microglia Cell Culture and Treatments

SIM-A9 (CRL-3265) mouse microglia cells were purchased from ATCC (Manassas, VA, United States) and were expanded in Corning^®^ T75 cm^2^ flasks with Dulbecco’s modified Eagle’s medium (DMEM high glucose 4.5 g/L, Caisson Labs, EE.UU, Utah) supplemented with 10% (vol/vol) 5% heat-inactivated horse serum and heat-inactivated fetal bovine serum 10% (Sigma Aldrich, EE.UU, Missouri), 50 units/ml penicillin, and 50 μg/ml streptomycin (Penicillin/Streptomycin, Sigma Aldrich, EE.UU, Missouri) in a 5% CO_2_ incubator at 37°C. The cells were split when they reached 70–90% confluence (plate ratio of 1:4) using a PBS/EDTA/EGTA/GLUCOSE solution (1 × /1 mM/1 mM/1 mg/ml) at 37°C for 1–5 min, followed by washing/resuspension in growth medium.

After confluence, cells were plated in 6-well plates and 96-well plates, at 200,000 and 5000 cells per well accordingly and pre-incubated with 5-Aza-2′-deoxycytidine (5-AZA) (Sigma Aldrich, A3656) or S-(5′-adenosyl)-L-methionine (SAM) (Sigma-Aldrich, A2408) for 24 h and cell viability was evaluated as described below. Based on cell viability assay in some experiments, we used preincubation of 250 μM SAM or 75 nM 5-AZA or 0.1% DMSO (Control) for 24 h followed by 500 ng lipopolysaccharide (LPS) (stock 1 μg/ml) (Sigma-Aldrich, L3023) or 100 μM palmitic acid (PAL) (Sigma-Aldrich, P9767) stimulation for the next 6 and 24 h. PAL was first solubilized in DMEM containing 10% of free fatty acid bovine serum albumin and then administered in each well.

### Cell Viability Analysis

Cell survival was determined using the MTT (Cell proliferation kit I, Roche Diagnostics, Mannheim, Germany) following manufacturer instructions by adding MTT (3-[4,5-dimethylthiazol-2-yl]-2,5-diphenyl tetrazolium bromide) for 1 h at 37°C in a CO_2_ chamber. Cell viability was quantified at 570 nm wavelength. Results are expressed as percentage of MTT reduction relative to control cells treated with 0.1% DMSO.

### Quantitative Phagocytosis Assay in Microglia Cells

Phagocytosis in microglia cells was determined using the green fluorescent latex beads (Sigma, L1030-1ML), which were pre-opsonized in fetal bovine serum (FBS) (1:5 ratio) for 1 h at 37°C. Subsequently, the FBS with the beads was added to the wells to obtain a final concentration of beads = 0.1% (v/v) as previously reported in Lian et al. (2016). After the cell treatment described previously, cells were incubated with the beads during 6 h at 37°C. We also incubated the experiment at 4°C for 6 h as a negative control.

The percentage of phagocytosis of the microglia was analyzed using a flow cytometer (BD Acuri C6 Plus). After the 6-h incubation ended, the culture medium containing the beads was discarded and the cells were washed twice with sterile 1 × PBS at 37°C. Cells were detached using 1 ml of the PBS/EDTA/EGTA/GLUCOSE solution (1 × /1 mM/1 mM/1 mg/ml) and resuspended in 100 μl of 1 × PBS for analysis in the BD Acuri C6 Plus flow cytometer in the channel FL1-A.

### Qualitative Phagocytosis Assay in Microglia Cells

To visualize phagocytosis under conditions of proinflammatory LPS and PAL stimulation and its modulation by 5-AZA and SAM methylation modulators, we used confocal microscopy. SIM-A9 cells were seeded on sterile coverslips in six-well plates covered with 0.01% poly-L-lysine solution (Sigma, p4707). After treatment, cells were fixed by adding 4% paraformaldehyde (PAF) (Sigma, 158127) for 10 min, washed three times with 1 × PBS, and were permeabilized with PBS solution 1 × + 0.1% Triton X-100 (PBST) (Sigma, × 100) for 10 min. Next, the cells were blocked in PBST + 10% goat serum (GIBCO, 16210064) for 30 min and washed three times with 1 × PBS. Microglia immunodetection was performed by overnight primary antibody anti-mouse Iba-1 (1:200) (Abcam, ab178847) incubation at 4°C followed by goat anti-rabbit IgG coupled to Alexa Fluor 546 (1:1000) (Invitrogen, A-11035). Finally, cells were washed 3 × with 1 × PBS and mounted in VECTASHIELD mounting medium with DAPI (Vector Laboratories, H-1000-10). Fluorescent signals were detected by confocal-laser microscopy using an Olympus BX61W1 microscope with an FV1000 module with diode laser. Finally, the images were processed with ImageJ software.

### NAc Shell and PFC Isolation and DNA/RNA Extraction

Brains were isolated from addiction-like and non-addiction-like behavior of subjects from the three dietary groups: chow diet, CAF, and CAF + Met and were frozen on dry ice and stored at −80°C until the collection of tissue. NAc shell and *PFC* of each brain were surgically isolated following stereotaxic coordinates according to Paxinos and Watson (2007) and total DNA/RNA extraction was performed by utilizing a purification kit (Qiagen) according to the manufacturer’s instructions.

### Quantitative Real-Time RT-PCR

We used 200 ng of total RNA for cDNA retro transcription using Applied Biosystems^TM^ High-Capacity cDNA Reverse Transcription Kit. Quantitative real-time RT-PCR was performed on a Lightcycler 480 system (Roche) using a iQ^TM^ SYBR^®^ Green SuperMix (Bio-Rad) with cDNA and primers (0.5 μM). The sequence primers used for analysis were as follows: IL-6 forward, 5′-TAGTCCTTCCTACCCCAATTTCC-3′. IL-6 reverse, 5′-TTGGTCAGCCACTCCTTC-3′. IL-1β forward, 5′-GCAACTGTTCCTGAACTCAAC-3′. IL-1β reverse, 5′-ATCTTTTGGGGTCCGTCAACT-3′. IL-1α-forward, 5′-GCACCTTACACCTACCAGAGT-3′. IL-1α reverse, 5′-TGCAGGTCATTTAACCAAGTGG-3′. TNFα forward, 5′-CAGGCGGTGCCTATGTCTC-3′. TNFα reverse, 5′-CGATCACCCCGAAGTCAGTAG-3′. 36B4 forward, 5′-TCCAGGCTTTGGGCATCA-3′. 36B4 reverse, 5′-CTTTATCAGCTGCACATCACTCAGA-3′. Changes in gene expression were evaluated using the −ΔΔCt method.

### Global DNA Methylation Analysis

Genomic DNA (50 ng/μl) was obtained as described before and quantified using the SYBR Green I protocol (Gragene, DNA Genotek Inc., Canada). For the global methylation analysis (Shiratori et al., 2016), 800 ng of DNA was digested with *Hpa*II (H) and with *Msp*I (M) restriction enzymes and incubated at 37°C for 2 h. Afterward, samples were incubated with the final extension reaction (1.7 × PCR buffer, 0.75 U Taq DNA polymerase) (Thermo Fisher Scientific) and 17 nM of TAMRA-dCTP (Jena Bioscience) for 1 h at 58°C in complete darkness. Aliquots of each final extension reaction were placed in 384-well black plates (Greiner Bio-One). The incorporation of TAMRA-dCTP fluorescence was directly measured using the Infinite M1000-Tecan microplate reader (excitation/emission 535/590 nm). Fluorescence polarization values (FP) were computed by the i-control^TM^ software (Tecan). The average of the FP values was calculated for each condition: FPSD, FPH, and FPM. The FPH and FPM values were normalized by subtraction of the FPSD value. Once the FP values of each sample were normalized, the 5 mC content of the genomic DNA was obtained with the following formula:% 5 mC ADN = (1-(FPH/FPM)) × 100.

### Statistical Analyses

All statistical tests were performed using the GraphPad Prism Version 7. For the *in vivo* data including the real-time RT-PCR analysis, we used one-way ANOVA, followed by a Bonferroni’s *post hoc* test. The differences between the groups of operant training test were tested by two-way repeated measures ANOVA. For the *in vitro* data including the real time RT-PCR and phagocytosis analysis, we used one-way ANOVA, followed by a Tukey’s *post hoc* test from one independent experiment of a total of four. Data are presented as mean ± SEM. The significance levels displayed on figures are as follows: ^∗^*p* < 0.05, ^∗∗^*p* < 0.01, ^∗∗∗^*p* < 0.001.

## Results

### Maternal Programming by Cafeteria Diet Favors Addiction-Like Behavior in Offspring

We initially evaluated the effect of cafeteria nutritional programming on addiction-like behavior in the offspring using the operant training response to get a cafeteria-like precision pellet. We identified responders to operant training based on number of lever presses during the FR1 and FR5 protocols. Analysis of self-administration during the FR5 schedule did not show effect on the total population of the first filial generation of offspring (F1) subjects from the cafeteria diet exposure during programming when compared with F1 control diet subjects (two-way repeated measures ANOVA, *p* < 0.001) (Supplementary Figure S1A). Data generated from the PR schedule also confirms that cafeteria programming does not affect lever presses responses in the F1 subjects (Supplementary Figure S1B). We previously reported that nutritional programming with CAF diet sets a low and high response to operant training for natural rewards during FR5 and PR schedules (Camacho, 2017). To diagnose addiction-like and non-addiction-like behavior phenotypes in the offspring subjects, we calculated the intake and motivation scores by integrating lever presses during the FR5 and PR schedules, respectively, as reported (Bock et al., 2013; Le et al., 2017). We use the $\times =\frac{X_{i}-\bar{X}}{s.d}$, where *X*_i_ is the behavior value for each rat, $\bar{X}$ is the mean behavior value for all the rats, and *s.d.* is the standard deviation of the population behavior value. We identified addiction-like and non-addiction-like behavior in the offspring by the FR5/PR ratio by setting a +0.5 or −0.5 score at “*x*” and “*y*” axis to characterize the addiction or non-addiction-like behavior subjects, respectively. Initially, we found that maternal programming by cafeteria diet exposure increases the number of F1 subjects diagnosed as addiction-like behavior when compared with control chow diet (chow = 11 vs. CAF = 14) (Figures 1B,C). Characterizing the F1 offspring showing major addiction-like behavior phenotype, we found that subjects of mothers exposed to CAF diet (*n* = 10) showed significant response to operant training schedule displaying high lever responses when compared to high responders (*n* = 10) of chow-exposed mothers (Figure 1D). No changes in lever responses were found in the non-addiction-like behavior subjects programmed by CAF or chow diets. This evidence confirms that caloric nutritional intake during pregnancy seems to modulate susceptibility to maximize operant responses to palatable food in offspring.

### Methyl-Donor Diet Reverts Addiction-Like Behavior in Offspring

**FIGURE 2:**
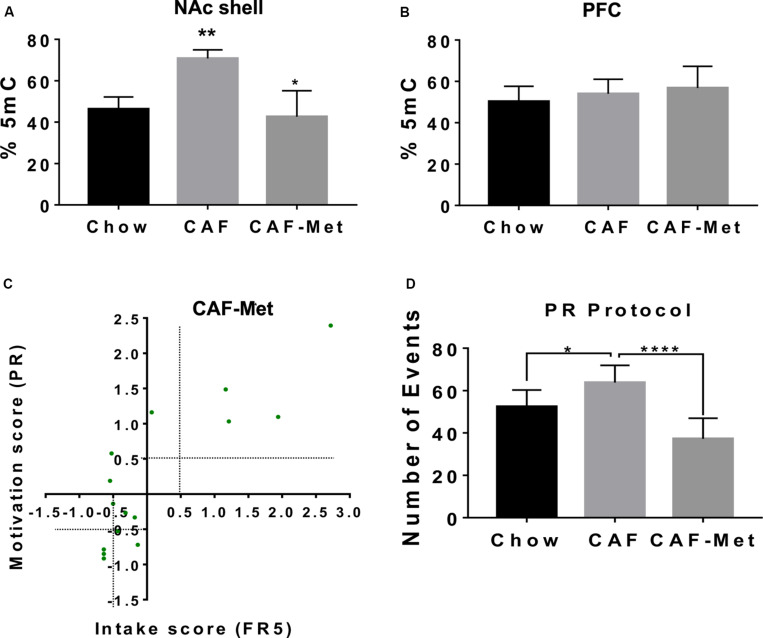
Methyl donors revert addiction-like behavior in the offspring. We fed female Wistar rats for 9 weeks as described with CAF + methyl donors (CAF + Met, *n* = 5). Offspring was fed with control diet and was trained in the operant training test protocol. **(A,B)** Global DNA methylome in NAc shell and PFC were identified using fluorescence polarization. Molecular characterization of pro-inflammatory expression and global DNA methylome in the NAc shell were performed in non-addiction and addiction-like behavior subjects. Graphs show mean ± SEM. Statistical significance after using one-way ANOVA, followed by a Bonferroni’s *post hoc* test. The differences between the groups of operant training test were tested by two-way repeated measures ANOVA. *p < 0.05 for CAF vs. CAF-Met; **p < 0.01 for CAF vs. chow. **(C)** Intake/motivation score for individual offspring subjects from mothers exposed to chow or CAF + Met diet followed the × = (×i–×^−−^)/(s.d) analysis (chow, *n* = 10; CAF, *n* = 14; CAF-Met, *n* = 4). **(D)** Number of events during PR protocol of subjects exposed to chow or CAF or CAF + Met diet during programming. Graphs show mean ± SEM. Statistical significance after using one-way ANOVA, followed by a Bonferroni’s *post hoc* test. The differences between the groups of operant training test were tested by two-way repeated measures ANOVA. *p < 0.05 for CAF vs. chow; ****p < 0.0001 for CAF vs. CAF-Met.

Maternal programming by external stimuli actively modulates epigenetic landscape, including the DNA methylome. We tested the effect of CAF diet exposure during programming on global DNA methylation and its modulation by methyl-donor supplementation [betaine (5 g/kg of diet), choline (5.37 g/kg of diet), folic acid (5.5 mg/kg of diet), and vitamin B12 (0.5 mg/kg of diet)] on addiction-like behavior. We found that programming by CAF diet actively increases global 5-methylcytosine DNA levels in the NAc shell and no changes were found in the PFC of offspring (Figures 2A,B). Unexpectedly, methyl-donor supplementation decreases 5-methylcytosine DNA levels similar to control values in the offspring (Figure 2A); however, it efficiently decreases the number of subjects displaying lever presses response on the FR5 and PR schedule (Supplementary Figures S2A,B). As before, we diagnosed the number of subjects showing addiction-like behavior diagnosed by the FR5/PR ratio by setting a +0.5 or −0.5 score at “*x*” and “*y*” axis to characterize the addiction- or non-addiction-like behavior. We identified that methyl donors decrease the addiction-like behavior displaying 14 offspring of CAF vs. 4 subjects included into the CAF-methyl donors (Figure 2C). Also, methyl donors efficiently decrease the number of events during the PR protocol (Figure 2D). These data propose that exposure to methyl groups during CAF programming decreases the 5-methylcytosine DNA levels in NAc shell and efficiently revert addiction-like behavior in the offspring.

### Methyl Supplementation to CAF Diet Increases Feed, Fasting, and Ghrelin-Sensitive Food Intake in Offspring

**FIGURE 3:**
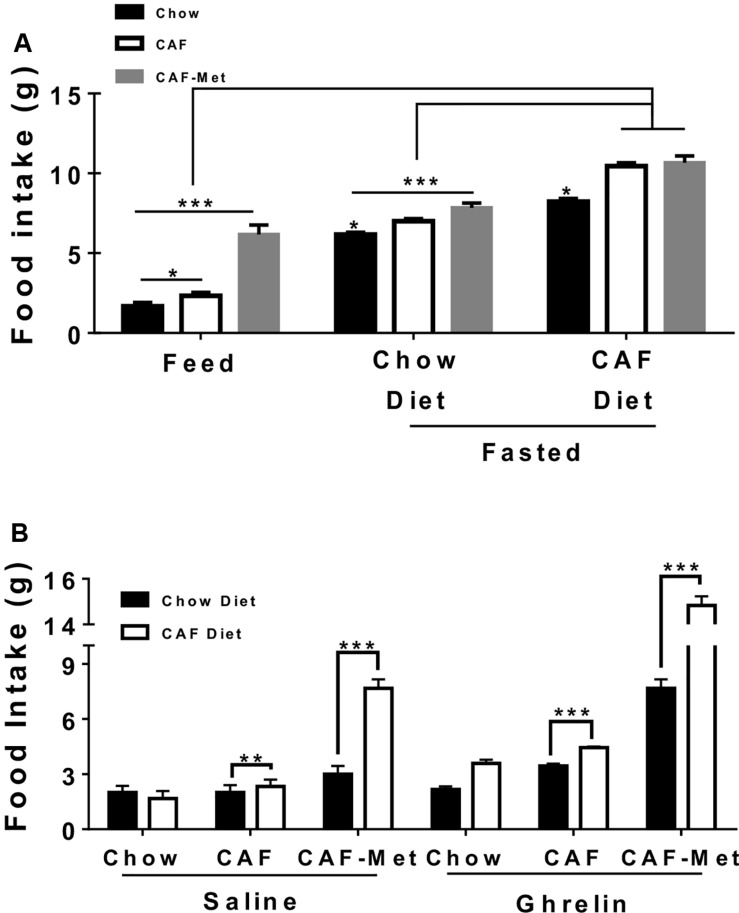
Effect of maternal nutritional programming on fed-fasting and ghrelin-sensitive food intake in offspring. **(A)** Maternal programming was performed by exposing chow or CAF or CAF + Met diet and daily food intake was quantified for each group (Feed state). Chow and CAF diet consumption during 4 h in offspring was registered after fasting for 16 h and refeeding. **(B)** Chow and/or CAF diet intake for 2 h after subcutaneous 0.2 μg/kg ghrelin administration (*n* = 10–12 per group). Graphs show normalized data of the mean ± SEM. Two-way ANOVA followed by Bonferroni multiple comparison test; *p < 0.05, **p < 0.01, ***p < 0.001.

Next, we identified the effect of maternal CAF and/or methyl-donor supplementation on physiological fast-feeding and ghrelin-sensitive food intake. We have recently reported that maternal programming by CAF diet primes ghrelin response to food intake in the offspring (Maldonado-Ruiz et al., 2019). Ghrelin has also been reported to modulate addiction-like behavior by its positive effects on the reward circuit. Our data initially showed that offspring from CAF diet-fed mothers showed increase in food intake of chow diet during feed and fasting scenarios, which is exacerbated when exposed to CAF diet (Figure 3A). Notably, programming by methyl donors leads to a substantial increase in CAF diet intake during the feed state, and a significant chow and CAF diet intake after fasting (Figure 3A). Also, methyl donors increase chow and CAF diet intake, up to twice and three times higher after intraperitoneal saline administration (Figure 3B). Remarkably, methyl donors promote a substantial increase up to three to five times of chow and CAF diet intake in the offspring following ghrelin systemic administration (Figure 3B). These results suggest that maternal programming by CAF diet actively promotes food intake by potentially priming ghrelin sensitivity in offspring, which is substantially exacerbated when exposed to methyl donors.

### Maternal Programming by CAF Diet Promotes Pro-inflammatory Expression in the NAc Shell of Addiction-Like Behavior Subjects

**FIGURE 4:**
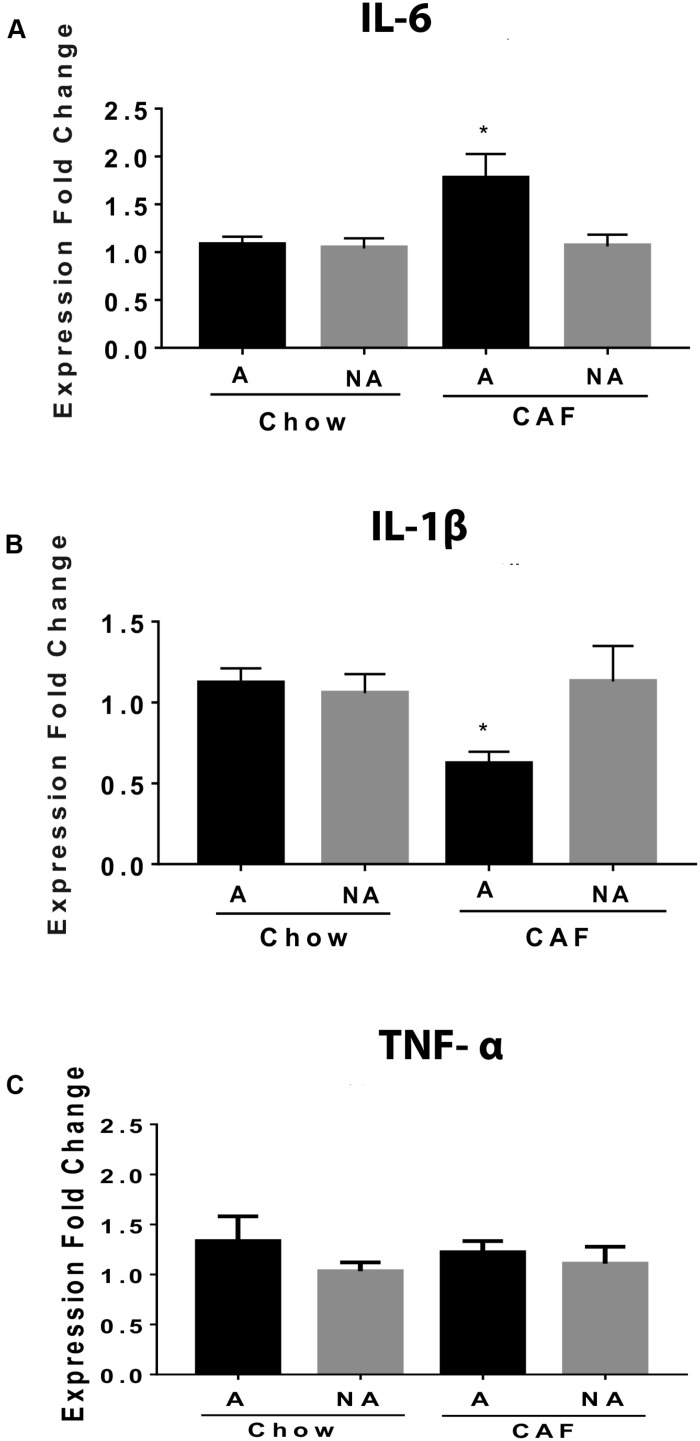
Maternal programming by CAF diet promotes IL-6 expression in the NAc shell of addiction-like behavior subjects. Maternal programming by chow or CAF diet exposure and addiction-like and non-addiction-like behavior diagnosis was performed as described. NAc shell was isolated following stereotaxic coordinates, total DNA/RNA was isolated by purification kit (QIAGEN), and RT-PCR analysis was performed by standardized methods. IL-6 **(A)** and IL-1β **(B)**, and TNF-α **(C)** mRNA expression was performed by selective primers and standardized methods. Graphs show normalized data of the mean ± SEM (*n* = 10–12 per group). Statistical significance after using one-way ANOVA, followed by a Bonferroni’s *post hoc* test. For IL-6, *p < 0.05 for A chow vs. A CAF; for IL-1β, *p < 0.05 for A CAF vs. A chow and NA CAF. A, Addiction-like; NA, Non-addiction-like.

We compared mRNA expression of proinflammatory cytokines involved in metabolic inflammation in subjects exposed to chow or CAF diets during programming. We found an increase in the IL-6 cytokine mRNA expression in the NAc shell of addiction-like behavior subjects programmed by CAF exposure when compared with non-addiction-like subjects or with chow-programmed F1 subjects (Figure 4A). Also, we found a decrease in the expression of IL-1β and no changes in TNF-α mRNA expression in the same addiction-like behavior of subjects programmed by CAF exposure when compared with non-addiction-like subjects or with chow-programmed F1 subjects (Figures 4B,C).

### Methylation Modulates Pro-inflammatory Cytokines Expression in Microglia Cells

**FIGURE 5:**
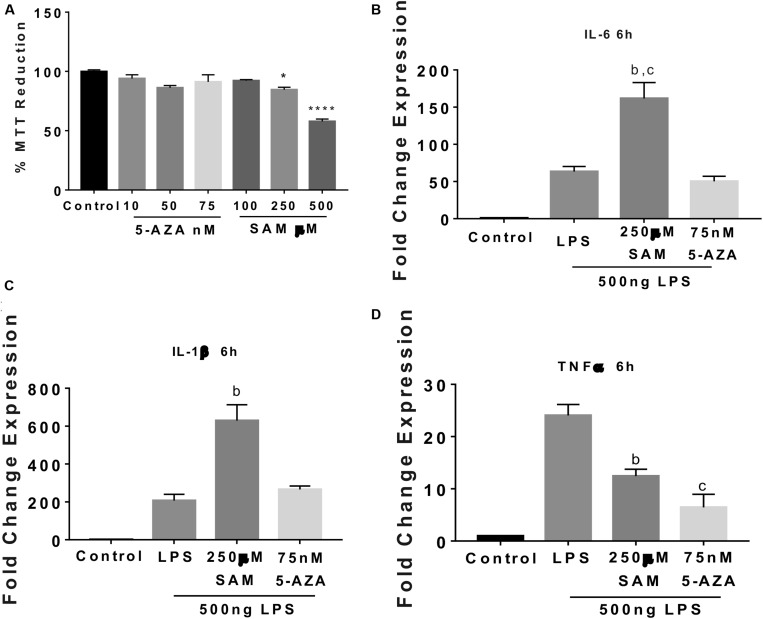
Methylation activates IL-6 and IL-1β gene expression followed by LPS stimulation in microglia cells. **(A)** Dose-dependent microglia cell viability during 24 h 5-AZA or SAM incubation was registered using standardized MTT protocols. Graphs show normalized data of the mean ± SEM for *n* = 4 and statistical significance after using one-way ANOVA with *post hoc* Tukey’s test. *p < 0.05, ****p < 0.0001. IL-6 **(B)**, IL-1β **(C)**, and TNF-1α **(D)** RT-PCR gene expression after 24 h 75 nM 5-AZA or 250 μM SAM pre-incubation followed by 6 h 500 ng of LPS (stock 1 μg/ml). Graphs show normalized data of the mean ± SEM relative quantification using 36B4 as a normalizing gene from one independent experiment of a total of four. Statistical significance after using one-way ANOVA with Tukey’s multiple comparisons test. For IL-6, ^b^*p* < 0.001 vs. LPS or vs. ^c^*p* < 0.0001 vs. 5-AZA; for IL-1β, ^b^*p* < 0.001 vs. 5-AZA, and for TNF-1α, ^b^*p* < 0.001 vs. LPS or vs. ^c^*p* < 0.0001 vs. LPS. 5-AZA, 5-Aza-2′-deoxycytidine; SAM, S-(5′-adenosyl)-L-methionine; PAL, palmitic acid.

Maternal programming by CAF exposure activates global DNA methylation, which correlates with an IL-6 increase and an IL-1β decrease in the NAc shell of addiction-like behavior subjects, we tested whether methylation/demethylation pharmacologic modulation promotes/reverts to a pro-inflammatory profile expression in the SIM-A9 microglia cells. Initially, we found a dose-dependent effect of 5-AZA or SAM on the MTT reduction showing no changes in cell survival at 75 nM 5-AZA and 250 μM SAM (Figure 5A). We did not find changes in IL-6, IL-1β, and TNF-1α gene expression followed by 5-AZA or SAM stimulation (data not shown). Real-time PCR analysis identified that favoring global DNA methylation by SAM incubation increases up to 292% and 200% IL-6 and IL-1β gene expression, respectively, following 6 h LPS incubation compared to LPS or LPS + 5-AZA treatments (Figures 5B,C). Conversely, active TNF-1α gene expression during LPS stimulation does not seem to depend on methylation/demethylation, given that SAM or 5-AZA incubation substantially downregulate TNF-1α gene activation up to 48% and 28%, respectively, when compared with LPS effect (Figure 5D).

**FIGURE 6:**
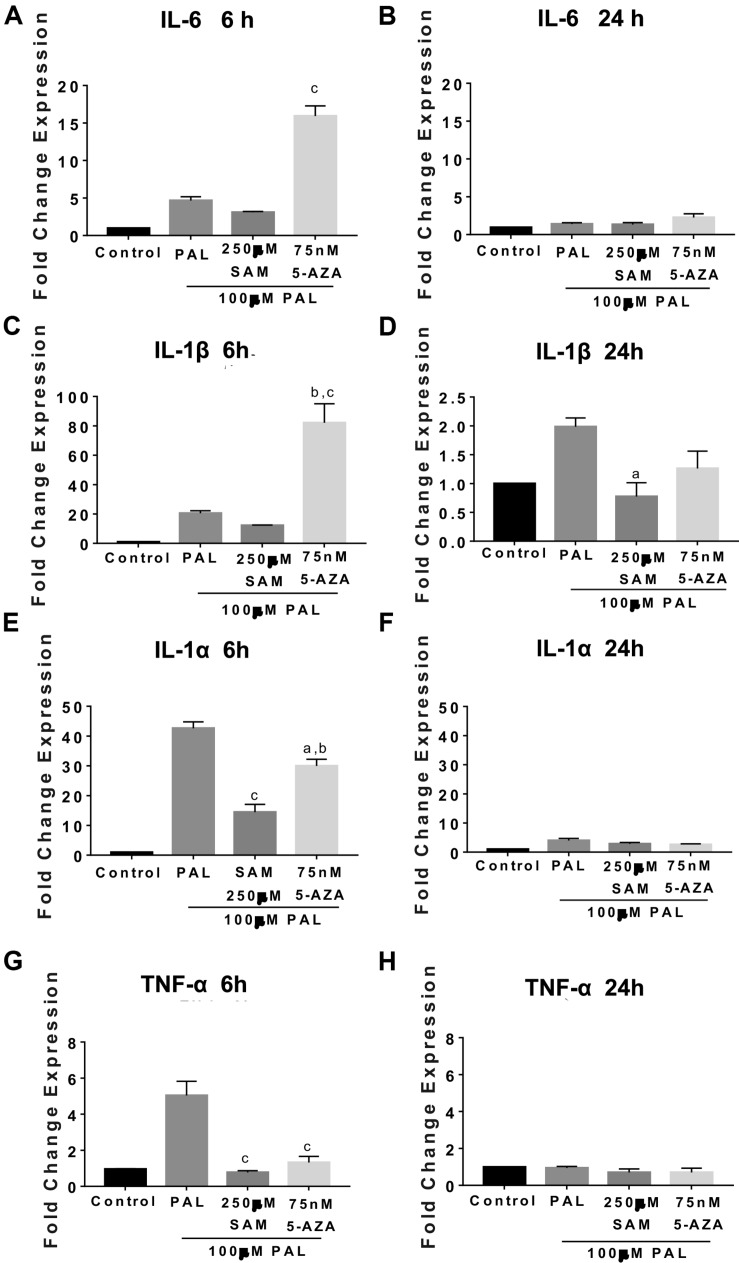
Demethylation activates IL-6 and IL-1β gene expression followed palmitic acid stimulation in microglia cells. Microglia cells were pre-incubated with 75 nM 5-AZA or 250 μM SAM for 24 h followed by 6 or 24 h stimulation of 100 μM PAL. IL-6 **(A,B)**, IL-1β **(C,D)**, IL-1α **(E,F)**, and TNF-1α **(G,H)** RT-PCR gene expression. Graphs show normalized data of the mean ± SEM relative quantification using 36B4 as a normalizing gene from one independent experiment of a total of four. Statistical significance after using one-way ANOVA with Tukey’s multiple comparisons test. For IL-6, ^c^*p* < 0.0001 vs. PAL and SAM; TNF-1α, ^c^*p* < 0.0001 vs. PAL; IL-1α ^a^*p* < 0.05 vs. PAL, ^b^*p* < 0.001 vs. SAM, and ^c^*p* < 0.0001 vs. PAL. 5-AZA. 5-AZA: 5-Aza-2′-deoxycytidine, SAM: S-(5′-adenosyl)-L-methionine, PAL, palmitic acid. IL-1β, ^a^*p* < 0.05 vs. PAL, ^b^*p* < 0.001 vs. PAL, and ^c^*p* < 0.0001 vs. SAM.

We also evaluated the effect of methylation/demethylation on IL-6, IL-1β, and TNF-1α gene expression following PAL stimulation. We have reported that intraventricular PAL injection in rats or incubation in microglia cells promotes IL-6, IL-1β, and TNF-1α cytokine release in microglia cells (Maldonado-Ruiz et al., 2019). Also, we have found that maternal programming by CAF diet sets a hypothalamic microglia activation in the offspring (Maldonado-Ruiz et al., 2019). We found that 5-AZA-induced demethylation actively promotes IL-6 and IL-1β gene expression following 6 h of 100 μM PAL stimulation and no changes by 24 h when compared with PAL itself (Figures 6A–D). Of note, replicating the results found during LPS stimulation, IL-1 alpha and TNF-1α gene expression during 5-AZA or SAM and 100 μM PAL incubation do not seem to be mediated by methyl donors given that 5-AZA or SAM actively promotes a downregulation of gene activation by 6 h (Figures 6E,G). Again, these effects are reverted to 48% and 28% of the control values after 5-AZA or SAM pre-incubation, respectively, followed by 24 h of PAL stimulation (Figures 6F,H). These results confirm that demethylation primes IL-6 and IL-1β gene activation following PAL or LPS stimulation.

### Methylation Modulates Microglia Phagocytosis in Culture

**FIGURE 7:**
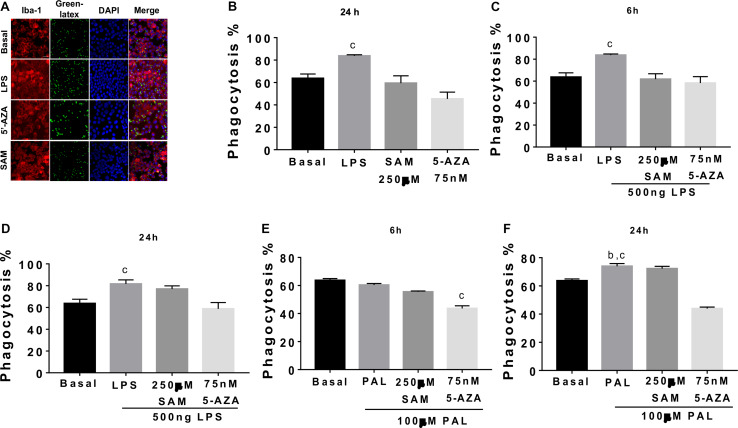
Demethylation inhibits microglia phagocytosis in microglia cells. **(A,B)** Microglia cells were pre-incubated with 75 nM 5-AZA or 250 μM SAM for 24 h, or LPS followed by pre-opsonized green fluorescent latex beads incubation for 1 h at 37°C. Qualitative phagocytosis was evaluated using immunofluorescence against anti Iba-1 (1: 200) followed by goat anti-rabbit IgG coupled to Alexa Fluor 546 (1:1000) and VECTASHIELD mounting medium with DAPI. Fluorescent signals were detected by confocal-laser microscopy using an Olympus BX61W1 microscope with an FV1000 module with diode laser and ImageJ software. ^c^*p* < 0.0001 for LPS vs. Basal, SAM and 5-AZA. **(C,D)** After 5-AZA or SAM incubation, microglia cells were treated with 500 ng of LPS for 6 or 24 h, respectively, and pre-opsonized green fluorescent latex beads were incubated as before.^c^*p* < 0.0001 for LPS vs. Basal, SAM + LPS and 5-AZA + LPS and ^c^*p* < 0.0001 for LPS vs. Basal and 5-AZA + LPS, respectively. **(E,F)** After 5-AZA or SAM incubation, microglia cells were treated with 100 μM PAL for 6 or 24 h, respectively, and pre-opsonized green fluorescent latex beads were incubated as before. Graphs show normalized data of the mean ± SEM relative to % phagocytosis of microglia cells from one independent experiment of a total of four. Statistical significance after using one-way ANOVA with Tukey’s multiple comparisons test. ^c^*p* < 0.0001 for PAL vs. 5-AZA + PAL; ^b^*p* < 0.001 for PAL vs. Basal and ^c^*p* < 0.0001 for PAL vs. 5-AZA + PAL.

Finally, we tested if methylation/demethylation potentially coordinates microglia phagocytosis following LPS or PAL incubation. Initially, we characterized a significant reduction of basal microglia phagocytosis followed by 5-AZA incubation when compared with basal microglia phagocytosis, LPS, or following SAM-induced methylation (Figures 7A,B). As expected, LPS stimulation efficiently activates microglia phagocytosis, which is blocked by 5-AZA demethylation induced at 6 and 24 h (Figures 7C,D). Also, methyl donors by SAM incubation are capable of preventing LPS-induced phagocytosis at least by 6 h and no effect at 24 h (Figures 7C,D). On the other hand, PAL shows a significant phagocytosis activation at 24 h, which is also reverted by 5-AZA pre incubation (Figures 7E,F). No changes in microglia phagocytosis were identified by SAM incubation followed by PAL stimulation (Figures 7E,F).

## Discussion

We report that maternal programming by exposure to hypercaloric diet elicits significant motivation to work for food evidenced by positive lever press responses to caloric pellets in the offspring. Notably, we found that hypercaloric diet intake promotes global DNA methylation in the NAc shell of subjects with addiction-like behavior, which correlates with an upregulation of IL-6 gene expression. We confirm that methyl donor’s exposure during programming efficiently decreases addiction-like behavior for caloric pellets in offspring, but instead, they showed a substantial ghrelin-sensitive response for food intake. These results suggest that maternal programming by hypercaloric diet selectively modulates the NAc shell methylome, favoring IL-6 gene expression, which is associated with incentive motivation for natural rewards and ghrelin-sensitive food intake response in the offspring.

In this study, we contribute to characterize the detrimental effect of maternal programming by caloric diets on incentive motivation behavior for food in the offspring. We identified that caloric overnutrition during pregnancy and lactation increases the number of offspring subjects that show addiction-like behavior for caloric pellets when compared with the offspring from mothers exposed to chow diet. Our results agree with previous reports confirming a hedonic phenotype to natural and non-natural rewards in the programmed offspring (Ong and Muhlhausler, 2011; Peleg-Raibstein et al., 2016; Sarker et al., 2018). Of interest, under FR1, FR5, and PR schedules, we identified two responder groups of subjects that displayed low and high motivation for lever presses, which are also identified under cocaine response (Verheij and Cools, 2011; Yamamoto et al., 2013), suggesting that susceptibility among individuals to addiction or external choices depends on genetic, epigenetic, and environmental factors (Pierce et al., 2018). Of note, motivation to work for hypercaloric food during the PR schedule showed a higher scale-up response in the offspring programmed by CAF diet, such as that reported for cocaine, alcohol, nicotine, heroin, and methamphetamine in rats (Peleg-Raibstein et al., 2016). It is important to mention that chronic food restriction in rats actively increases dopamine levels in NAc, leading to augmentation of hedonic behavior (D’Cunha et al., 2017). We used a food restriction protocol in order to enhance the lever presses response during the FR1 schedule; however, we evaluated the hedonic phenotype for natural rewards in the FR5 and PR schedules (4 and 7 days after the *ad libitum* feeding schedules). This protocol allows us to selectively characterize the effect of maternal programming on hedonic rather than metabolic parameters. In line with this evidence, humans have shown increased caloric food liking and/or increased hunger ratings in subjects classified as food addicts (Loxton and Tipman, 2017; Ruddock et al., 2017). Mechanistically, it has been proposed that excessive high-energy food consumption seems to negatively modulate a hedonic phenotype in animal models (Kenny, 2011; Barry et al., 2018; Ducrocq et al., 2019), in part, by downregulating the D2 dopamine receptors (Johnson and Kenny, 2010; Winterdahl et al., 2019), and/or reduced dopamine release (Avena and Bocarsly, 2012). In our context, it seems that maternal programming by caloric exposure and/or food restriction primes a new state of reward deficiency integrating lower availability dopamine receptors and dopamine release, which might favor impulsiveness for unhealthy eating in order to cope against deficient dopaminergic neurotransmission (Dietrich et al., 2014; Bongers et al., 2015). Taken together, our work reaffirms the proposal that fetal programming by hypercaloric diet in mothers favors motivating behavior toward natural high caloric value rewards in the offspring (Claycombe et al., 2015; Edlow, 2017), which might replicate major hedonic value for food in subjects classified as food addicts (Loxton and Tipman, 2017; Ruddock et al., 2017).

Here, we reported for the first time that CAF diet exposure during pregnancy and lactation leads to selective global DNA methylation in the NAc shell and no changes in the PFC of addict offspring subjects. DNA methylation is a deep coordinator of fetal programming by assisting selective developmental states capable of modulating long-lasting changes in brain plasticity related to motivation addiction (Honegger and de Bivort, 2018). In our maternal programming model, pregnant females are expected to have uneven litter load during gestation and might be exposed to differential fetal programming, which denotes a potential limitation of the murine model. However, litters were adjusted to the same number in the chow and CAF experimental groups. Molecular epigenetic signatures for setting food addiction in the offspring are not totally understood because food is a natural and necessary reward for animals and humans when compared with drugs or alcohol. For example, changes in DNA methylation could be responsible for neuroplasticity changes induced by addictive drugs, such as repeated cocaine administration reduced global levels of H3K9 dimethylation in the NAc, which correlates with changes in dendritic spines (Maze et al., 2010). Also, changes of the histone dimethyltransferase G9a in the NAc shell actively affect cocaine self-administration in rats (Anderson et al., 2018, 2019). Of importance, DNA methylation in NAc during drug addiction seems to respond to a time-dependent regulation, enhancing DNA methylation in the NAc after 30 days of cocaine withdrawal (Massart et al., 2015), suggesting that an increase in DNA methylation is responsible for cocaine craving. In agreement with these data, we found that programming by CAF diet substantially increases global DNA methylation in the NAc shell of addict offspring that are no longer exposed to CAF diet. Recent evidence have identified that CAF programming promotes D2 dopamine receptor DNA methylation in NAc of the offspring (Rossetti et al., 2020), which potentially might explain the impulsiveness for unhealthy eating. Notably, methyl donors revert the percentage of global DNA methylation and block addiction-like behavior in the offspring. Our data agree with the fact that chronic treatment with the methyl-donor methionine inhibits cocaine-induced conditioned place preference in rats (Tian et al., 2012), which seems to depend on cocaine-induced c-Fos expression in the NAc (Wright et al., 2015). Also, it has been reported that fetal programming by high-fat diet promotes global DNA hypomethylation in the offspring reward circuit (Skinner et al., 2011; Carlin et al., 2013), and in fact, it is also detected in response to fetal programming by environmental exposure to toxins (Baccarelli and Bollati, 2009), during restriction of nutrients *in utero* or prenatal exposure to cocaine (Novikova et al., 2008). In addition, global hypomethylation correlates with an increased expression of opioid receptors and the dopamine transporter in the NAc of the high-fat diet-programmed offspring (Carlin et al., 2013). Conversely, maternal diet supplementation with methyl donors seems to be beneficial by restoring weight gain and food intake in animals exposed to caloric diet (Carlin et al., 2013). Alternatively, methyl-donor supplementation during programming might also contribute to additional metabolic or systemic molecular pathways away to the effects in DNA methylation during neonatal development. For instance, methyl-donor infusion in piglets increases creatine, whereas it decreases phosphatidylcholine levels in liver (Robinson et al., 2016). Creatine synthesis itself requires glycine and an amidino group (provided by arginine) and a methyl group (provided by SAM) (Brosnan et al., 2009), supporting the notion that methyl donors modulates amino acids metabolism in energy-demand tissues including brain. Finally, in a remarkable report, Rizzardi et al. (2019) identified 12 Mb of differentially methylated CG regions in flash-frozen postmortem human tissues including dorsolateral PFC, hippocampus, NAc, and anterior cingulate gyrus implicated in neuropsychiatric disorders, including addiction (Rizzardi et al., 2019). Our data suggest a differential and opposite effect of drugs and food on DNA methylation in NAc; however, in both scenarios, NAc methylation favors addiction-like behavior.

One of the major results from our study proposes that maternal programming by methyl donors decreases the incentive to work for natural rewards; however, it does prime a metabolic aberrant phenotype promoting overfeeding during fasting and ghrelin-sensitive schedules. We have previously reported that CAF diet primes a fasting and ghrelin-sensitive food intake response in the offspring when compared with the offspring of mothers exposed to chow diet (Maldonado-Ruiz et al., 2019). Overfeeding in the programmed offspring might be explained by two scenarios, firstly, postnatal high caloric diet exposure has been identified to induce hypermethylation of the Pomc promoter, blocking leptin signaling and favoring food consumption and weight gain (Marco et al., 2013; Zhang et al., 2014). In fact, higher methylation levels at the proopiomelanocortin (POMC) CpG sites +136 bp, and +138 bp and lower methylation of the neuropeptide Y promoter in human leukocytes have been associated to weight gain (Crujeiras et al., 2013). A potential second scenario might be related with hypomethylation in the neuropeptide Y promoter (Lazzarino et al., 2017) favoring overfeeding after fasting or ghrelin administration in the offspring. We have reported that fetal programming by CAF diet exposure exacerbates hypothalamic c-fos neuronal response to ghrelin in the offspring (Maldonado-Ruiz et al., 2019), suggesting that DNA methylation during maternal CAF exposure might disrupt the expression of food intake neuropeptides favoring ghrelin-sensitive orexigenic signaling.

Next, we characterized the molecular immune signatures of fetal programming in the offspring that favor or correlate with addiction-like behavior. We have reported that maternal programming by CAF exposure leads to hypothalamic microglia activation in the offspring (Maldonado-Ruiz et al., 2019). Our *in vivo* data show a higher expression of IL-6 in the NAc shell of subjects that exhibited addiction-like behavior. Our *in vitro* experiments indicate that LPS or PAL incubation efficiently activates TNF-α, IL-6, and IL-1α gene expression. Also, methyl-donor incubation activates or decreases IL-6 and IL-1α gene expression following LPS or PAL stimulation, respectively. Our results agree with recent reports demonstrating that 5-AZA-induced demethylation significantly increases IL-6 and IL-8 gene expression in human dental pulp cells (Mo et al., 2019) and in polymorphonuclear cells (Shen et al., 2016) followed by LPS stimulation. Potentially, we propose that DNA methylation might coordinate a pro- and anti-inflammatory profile in macrophages toward both M1 and M2 phenotypes. For example, LPS efficiently leads to SOCS1 gene promoter methylation in macrophages favoring secretion of TNF-α and IL-6 cytokines (Cheng et al., 2014). Global DNA hypermethylation has also been reported in inflamed tissues of obesogenic-diabetic mellitus mice models, showing an M1 phenotype in macrophages (Babu et al., 2015; Kapellos and Iqbal, 2016). In our context, positive energy balance such as the obesogenic-diabetic mellitus mice models are closely related to the metabolic failure found in our maternal programming model (Cardenas-Perez et al., 2018), suggesting that global DNA methylation in NAc might potentially be associated to positive energy balance. Finally, our *in vitro* experiments identified that demethylation selectively blocks phagocytosis in microglia during LPS or PAL stimulation. There are no available studies regarding the effect of DNA methylation/demethylation on phagocytosis in microglia, which allow us to integrate this mechanism on addiction behavior. A previous study reported 5-AZA-induced demethylation and significantly increased efferocytosis in alveolar macrophages from COPD patients (Barnawi et al., 2017), which potentially reflects a pathological scenario linked to immunity.

We propose that maternal programming increases susceptibility to addiction-like behavior in the offspring integrating global DNA methylation in the NAc shell, which correlates with IL-6 gene expression. CAF diet made of lipids such as PAL might imitate foreign compounds and initiate a proinflammatory immune signaling in the brain to create its rewarding and/or overfeeding effects.

## Data Availability Statement

All relevant data are contained within the manuscript.

## Ethics Statement

The animal study was reviewed and approved by the Local Animal Care Committee (BI0002) at the Universidad Autónoma de Nuevo León, Mexico.

## Author Contributions

GC-C, LM-M, RM-R, MC-T, SB-V, and AC-M: conceptualization. GC-C, LM-M, MC-T, SB-V, RM-R, DR-P, DR-R, GL, LG-O, and AC-M: investigation. GC-C, LM-M, RM-R, MC-T, SB-V, DR-R, GL, and AC-M: methodology. DR-P, GL, LG-O, and AC-M: supervision and visualization. GC-C, LM-M, RM-R, MC-T, DR-P, DR-R, GL, LG-O, and AC-M: writing – review and editing.

## Conflict of Interest

The authors declare that the research was conducted in the absence of any commercial or financial relationships that could be construed as a potential conflict of interest.
