# Supplementary material for: Fetal Programming by Methyl Donors Modulates Central Inflammation and Prevents Food Addiction-Like Behavior in Rats
Source: Front Neurosci. 2020 Jun 3;14:452. doi: 10.3389/fnins.2020.00452 (PMC7283929; doi:10.3389/fnins.2020.00452)
Supplement: Supplementary file 1 [file Table_1.DOCX]

**Supplemental Information**

Fetal programming by methyl donors modulates central inflammation and prevents food addiction-like behaviour in rats

Gabriela Cruz-Carrillo, Larisa Montalvo-Martínez, Marcela Cárdenas-Tueme, Sofia Bernal-Vega, Roger Maldonado-Ruiz, Diana Reséndez-Pérez , Gertrud Lund, Lourdes Garza-Ocañas and Alberto Camacho-Morales.


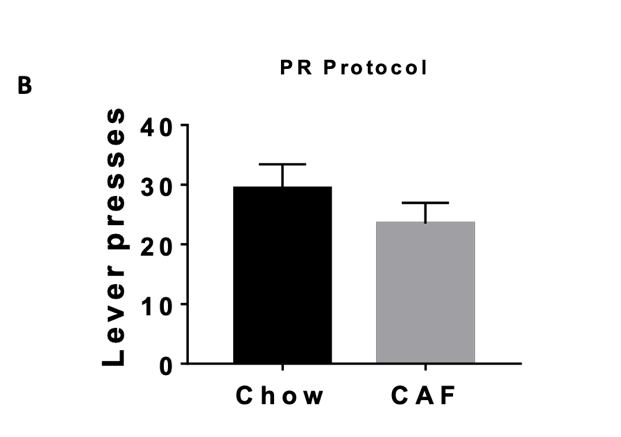
Supplemental figures


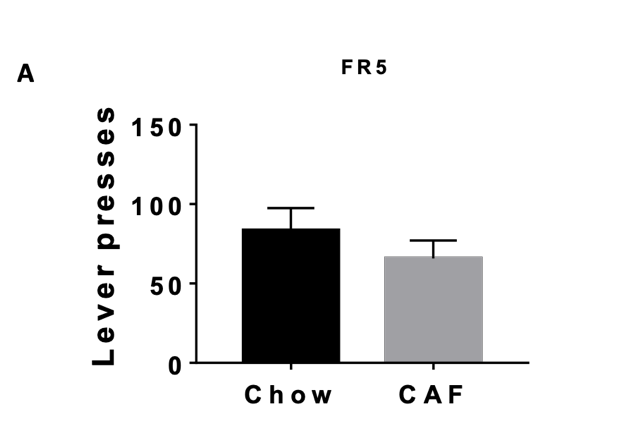


*Figure S1. Maternal programming by CAF diet primes addiction like behaviour in offspring. Offspring was fed with control diet after weaning until 2 months of age and was trained in the operant training test protocol to diagnosed non-addiction and addiction-like behaviour. Number of lever presses during the FR5 protocol (A) or PR protocol (B) of offspring. Graphs show mean ± S.E.M. Chow n = 53, CAF n=36. Unpaired Student’s t-test.*

**

*Figure S2. Maternal programming by CAF-Met diet reverts addiction like behaviour in offspring. Offspring was fed with control diet after weaning until 2 months of age and was trained in the operant training test protocol to diagnosed non-addiction and addiction-like behaviour. Number of lever presses during the FR5 protocol (A) or PR protocol (B) of offspring. Graphs show mean ± S.E.M. Chow n = 36, CAF-Met n=19. Unpaired Student’s t-test. *p < 0.05,**p < 0.01.*

*Table 1. Fiber available per 100 g of Chow and CAF diet*

**
